# Nutritional 1C Imbalance, B12 Tissue Accumulation, and Pregnancy Outcomes: An Experimental Study in Rats

**DOI:** 10.3390/nu10111579

**Published:** 2018-10-26

**Authors:** Ole Nymark, Ebba Nexo, Eva Greibe

**Affiliations:** Department of Clinical Biochemistry, Aarhus University Hospital, Palle Juul-Jensens Boulevard 99, 8200 Aarhus N, Denmark; au335591@post.au.dk (O.N.); ebbanexo@rm.dk (E.N.)

**Keywords:** vitamin B12, cobalamin, cyano-B12, hydroxo-B12, pregnancy, rats, 1C metabolism

## Abstract

Vitamin B12 deficiency during pregnancy has been associated with poor fetal outcome. Here we investigate the influence of a one-carbon (1C) imbalanced diet (low B12, high folate, high methionine) on maternal B12 status, fetal outcome, B12 distribution, and on the 24-h distribution of synthetic cyano-B12 (CN-B12) and natural hydroxo-B12 (HO-B12). Female Wistar rats were mated while on a 1C balanced (*n* = 12) or imbalanced diet starting two weeks (*n* = 10) or four weeks (*n* = 9) prior to pregnancy and continuing throughout pregnancy. At gestation day 18 (out of 21), all rats received an oral dose of labeled CN-B12 or HO-B12. After 24 h, the rats were sacrificed. Fetuses were inspected, and maternal tissues and fetuses were measured for endogenous and labeled B12. Pregnancy caused a redistribution of B12 from the kidneys to the liver and fetal compartment (uterus, placenta, fetuses). The 1C imbalanced diet reduced maternal kidney B12 and gave rise to lower-weight fetuses with visual malformations. In contrast, fetal B12 did not reflect fetal outcome. This suggests that maternal B12 is more important for fetal outcome than fetal B12. The 24-h distribution of labeled B12 in the rats on the 1C imbalanced diet showed a higher fetal accumulation of CN-B12 than HO-B12, while the opposite was seen in the maternal tissues.

## 1. Introduction

Vitamin B12 (B12) is an essential coenzyme important for fetal development. Maternal B12 deficiency during pregnancy has been associated with poor fetal outcomes such as intrauterine growth retardation, recurrent fetal loss, low birth weight, and neural tube defects [1,2,3,4,5]. Yet, a rare group of patients presents a paradox. Children with inborn errors in B12 trafficking (transcobalamin deficiency) or in intracellular B12 metabolism (CblA-G) are often born normal, in spite of the defective B12 machinery during embryogenesis [6]. Within a few days/weeks after birth, the children develop life-threatening B12 deficiency, which must be treated to prevent permanent damage, including movement disorders and cognitive impairment [7]. For unidentified reasons, injection therapy with hydroxo-B12 (HO-B12), a natural B12 form present in food, ensures better recovery than treatment with cyano-B12 (CN-B12), the synthetic B12 form commonly used in supplements [7]. Our recent rat studies may present at least a partial explanation. We find that CN-B12 and HO-B12 are handled differently in the body [8,9,10], and that HO-B12 provides more active B12 coenzymes, necessary for the B12 metabolism, to the tissues [10].

The placental B12 transport in rodents has been studied in the past using radiolabeled CN-B12 [11,12,13]. The studies indicate that the placenta accumulates B12 and can modulate the transfer of B12 to the fetal circuit in a rate-limited manner, possibly via a receptor-mediated mechanism analogous to the transfer of intrinsic factor-bound B12 across the ileal mucosa and into the blood [11,12]. The transfer from placenta to fetus occurs against a concentration gradient, as the fetal plasma B12 levels are much higher than the corresponding maternal levels in late pregnancy [11,12]. To the best of our knowledge, placental transport of radiolabeled HO-B12 has never been investigated. In the light of our recent findings that CN-B12 and HO-B12 are handled differently in the male rat [8,9,10], we set out to compare the maternal and fetal accumulation of CN-B12 as compared to HO-B12 during pregnancy. As B12 is part of the one-carbon (1C) cycle and is linked to the metabolism of other 1C metabolites, such as folate and methionine, we designed the study to investigate fetal outcome and maternofetal B12 accumulation in pregnant rats on a standard diet (balanced in 1C components) and on a 1C imbalanced diet depleted in B12 but with a high content of folate and methionine. We present data on: (1) the B12 accumulation in whole fetuses and selected maternal tissues (kidney, liver, brain, uterus, and placenta); (2) the distribution of newly administered CN-B12/HO-B12 in the pregnant rat and the fetus; and (3) the recorded fetal outcomes.

## 2. Materials and Methods

### 2.1. Animals

Female Wistar rats (RjHan:WI) (*n* = 31) (Janvier Labs, Le Genest-Saint-Isle, France*)* (seven weeks old, weighing approximately 200–300 g on arriving at the animal facility) were used in the experiment. The study was approved by the Danish Animal Ethics Committee Inspectorate in agreement with European Union directive 2010/63/EU on animal experiments (approval number: 2016-15-0201-00984) and was carried out at the animal facility at Health Faculty, Aarhus University, Denmark, in accordance with the ARRIVE guidelines. The institutional and national guidelines for care and use of animals were followed, and professional animal caretakers checked the rats daily for any health or welfare problems. No signs of pain, suffering, or distress were observed before or during the study.

The rats were housed in pairs in cages (Techniplast/Makrolon/1291 H type III H/800cm2, Techniplast, Buguggiate, Italy) in a controlled environment with a 12-h day and night cycle. The room temperature was 19–20 °C with 40–60% humidity, and the rats had free access to food and tap water. Bedding material (asp chips, Tapvei, Kortteinen, Finland) and soft paper wool (LBS biotech, Surrey, UK) were changed weekly. The rats were allowed to acclimatize for two weeks at the animal facility before the study was initiated.

### 2.2. Study Design

**Figure 1:**
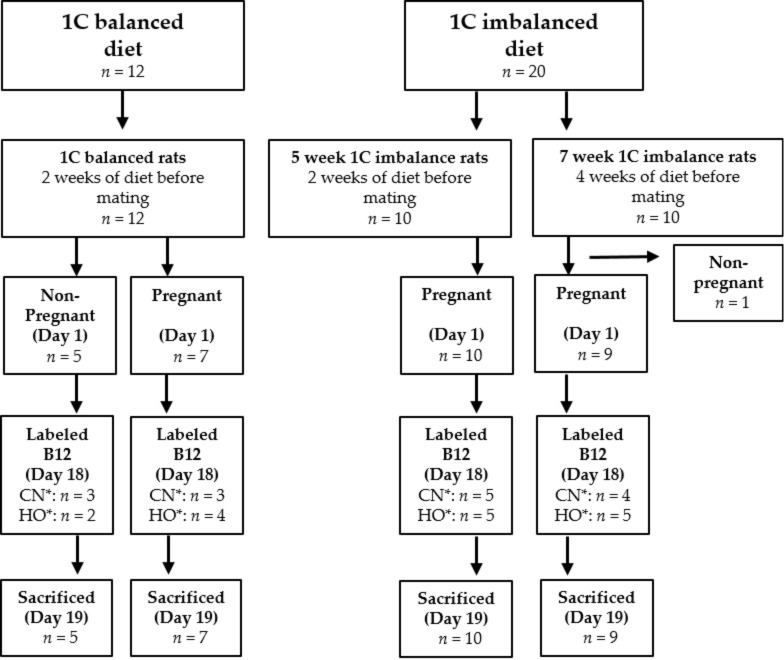
Study design. Abbreviations: CN*: [^7^Co]CN-B12, HO*: [^57^Co]HO-B12. 1C: one-carbon.

The experiment was conducted over a period of seven weeks (study design is outlined in Figure 1). The rats were impregnated while on a standard 1C balanced diet (*n* = 12) or a 1C imbalanced diet starting two or four weeks prior to pregnancy and continuing throughout the three-week pregnancy period. In total, these rats received the 1C imbalanced diet for five weeks (5-week 1C imbalanced, *n* = 10) and seven weeks (7-week 1C imbalanced, *n* = 9), respectively. Five rats did not become pregnant and continued in the study as controls (non-pregnant, five-week 1C balanced diet, *n* = 5) (Figure 1).

According to the manufacturer, the 1C balanced diet (a standard rat diet) (Altromin 1324, Brogaarden, Lynge, Denmark) contained 24 µg/kg B12, 2 mg/kg folate, and 0.3 mg/kg methionine. In contrast, the 1C imbalanced diet (Altromin C1024, Brogaarden, Lynge, Denmark) contained 11 µg/kg B12, 10 mg/kg folate, and 10,635 mg/kg methionine. The dietary content of components relevant for the 1C metabolism are shown in Table 1. For all details on the dietary compositions, see the manufactures website (www.brogaarden.eu).

For impregnation, female rats were paired two-and-two with male rats in so-called “sniffing cages” where rats are kept apart by a metal grid for three days to induce ovulation through olfactory stimulation. At day 3, the female and male rats were joined by removal of the grid to allow for mating within a period of 36 h. On day 1 and 19 (two days before term), the rats were weighed and blood samples were collected from the sublingual vein into lithium heparin tubes (BD Vacutainer) with a 23-gauge needle. Plasma was removed after centrifugation (9 min, 1850× *g*), and stored at −20 °C until analysis.

Twenty-four hours prior to sacrifice, the rats in the pregnancy groups (1C balanced, 5-week 1C imbalanced, and 7-week 1C imbalanced) and in the control group (non-pregnant) were randomly allocated to receive an oral dose of labeled B12, either [^57^Co]CN-B12 or [^57^Co]HO-B12 (see Section 2.4), by gastric gavage using a 20-gauge needle. After the 24 h, the rats were anesthetized with isoflurane gas and sacrificed by cervical dislocation. Liver, kidneys, brain (cerebrum), and fetal compartment (uterus, placentas, and fetuses) were collected, weighted, and snap-frozen in liquid nitrogen before stored at −80 °C until analysis (see Section 2.3). The decision to sacrifice the rats on gestation day 19 (two days before term) was made to ensure that none of the pregnant rats went into labor before the study was completed.

For practical reasons and due to a limited number of “sniffing cages”, the study with 1C imbalanced rats (May 2017) was done three months after the study with 1C balanced rats (February 2017), and the groups of 5-week 1C imbalanced and 7-week 1C imbalanced rats were impregnated two weeks apart. However, we put great efforts into ensuring that the study conditions (same room, temperature, humidity etc.) and handling (same operators, identical timeline etc.) were identical for all groups. We used the same batches of [^57^Co]CN-B12 and [^57^Co]HO-B12 for all groups and administered the same total dose of radioactivity (cpm) adjusted with unlabeled B12 to provide the same total amount of B12 to all rats (see Section 2.4 for details).

### 2.3. Endogenous B12

Endogenous plasma B12 was measured on the ADVIA Centaur CP immunoassay system, (Siemens Healthcare Diagnostics, Ballerup, Denmark), either undiluted (rats on the 1C imbalanced diet) or using a 1:2 dilution with 0.9% NaCl (rats on the 1C balanced diet).

Tissues were thawed on ice and the content of endogenous B12 was measured in extracts from 250 mg liver, kidneys, brain, uterus, and placenta. Four fetuses from each group were also selected for B12 analysis. The selection was done as follows: four rats from each group were selected based on total maternal B12 levels closest to the group median. From each litter, the fetus closest to the median fetus weight was chosen for B12 analysis. Each whole fetus was dissected into samples of 250 mg. To each sample (tissues or fetus), 750 µL of Na-acetate buffer (0.4 mol/L, pH 4.4) was added and the mixture was homogenized (6800 rpm of three cycles of 20 s with 30-s pauses in-between cycles) with the Precellys 24 (Bertin Technologies, Montigny-le-Bretonneux, France. After homogenization, 20 µL of KCN (30 mmol/L) was added, and the mixture was boiled for 10 min. Then, the mixtures were centrifuged for 40 min at 20,000× *g* and the supernatants were collected and stored a −20 °C until analysis. The supernatants were measured for total endogenous B12 content on the ADVIA Centaur CP Immunoassay System after dilution with 0.9% NaCl to ensure that the B12 concentrations would be within the measurement range (36–1464 pmol/L). The results are expressed as pmol B12 per gram of wet tissue and as whole-organ B12.

### 2.4. Twenty-Four Hour Uptake of Labeled B12

Twenty-four hours prior to sacrifice, the rats received an oral dose of 1 pmol labeled B12 (approximately 200,000 cpm) as either [^57^Co]CN-B12 or [^57^Co]HO-B12 in 0.5 mL water administered through gastric gavage as previously described [8,9,14]. Commercially available [^57^Co]CN-B12 (1.75 µCi/mL, MP Biomedicals, Solon, OH, USA, catalogue no. 06B-430000) was used. HO[^57^Co]-B12 was produced by converting CN[^57^Co]-B12 to HO[^57^Co]-B12 by photoaquation in an acidic medium under nitrogen bubbling [8] (a generous gift from Sergey N. Fedosov). The labeled B12 was adjusted to the desired concentration by addition of unlabeled CN-B12 (cyanocobalamin, Sigma-Aldrich, Brøndbyvester, Denmark) or HO-B12 (Vibeden, Sandoz, Copenhagen, Denmark). The term [^57^Co]B12 will be used throughout the text to cover both forms of labeled B12 ([^57^Co]CN-B12 and [^57^Co]HO-B12).

Accumulation of [^57^Co]B12 in fetuses and maternal tissue were assessed by counting of the whole organs for 1 min in a gamma counter (2470 Wizard^2^ Automatic Gamma Counter, Perkin Elmer, Skovlunde, Denmark) as described in [8,9,15]. In practice, the tissues were thawed on ice, cut into smaller pieces, and transferred to tubes for the gamma counter. All tubes were counted to obtain the whole-organ cpm. Tissue results are expressed as the fraction (%) of the total administered dose (cpm) of labeled B12 per organ. Plasma samples were counted for nine hours due to the low amount of cpm/mL. By increasing the counting time, we decrease the level of uncertainty because this is related to the total amount of cpm (uncertainty = √cpm/cpm). Plasma results are expressed as the fraction (%) of the total administered dose (cpm) of [^57^Co]B12 per mL plasma.

### 2.5. Statistical Methods

The number of animals in each pregnancy group was based on power calculations using a multiple linear regression model showing a statistical power of 90% and a two-sided significance level of 5%. As we did not know the dynamics of CN-B12 and HO-B12 during pregnancy, we based our sample size calculations on an earlier study showing a 73% decline in plasma B12 (from 1388 pmol/L to 378 pmol/L) in response to four weeks on the 1C imbalanced diet [10]. We wished to detect a decline of 60% in plasma B12. Sample size calculations suggested *n* = 3 in each subgroup ([^57^Co]CN-B12 and [^57^Co]HO-B12); however, to account for sick animals and for rats not becoming pregnant, we included at least five rats in each subgroup (see Figure 1).

The D’Agostino–Pearson omnibus test was used to test whether data followed the Gaussian distribution. Normally distributed data was compared using the one-way ANOVA with Tukey’s post hoc corrections for multiple comparisons and the unpaired *t*-test. Not normally distributed data was compared using the Kruskal–Wallis test with Dunn’s corrections and the Mann–Whitney test with Bonferroni correction. For paired data (blood over time), the paired *t*-test (normally distributed data) or the Wilcoxon signed rank test (not normally distributed data) was used. Values of *p* <0.05 were considered statistically significant unless otherwise stated. All data was analyzed using the software available by GraphPad Prism 7 for Windows, version 7.03, (GraphPad, La Jolla, CA, USA).

## 3. Results

We investigated fetal outcome and maternal tissue and fetal accumulation of endogenous B12 and newly-absorbed labeled B12 (CN-B12 and HO-B12) in rats on 1C balanced and imbalanced diet. We compared four groups of rats: non-pregnant 1C balanced rats (controls) and three groups of pregnant rats: 1C balanced, 5-week 1C imbalanced, and 7-week 1C imbalanced.

### 3.1. Basic Characteristics

**Figure 2:**
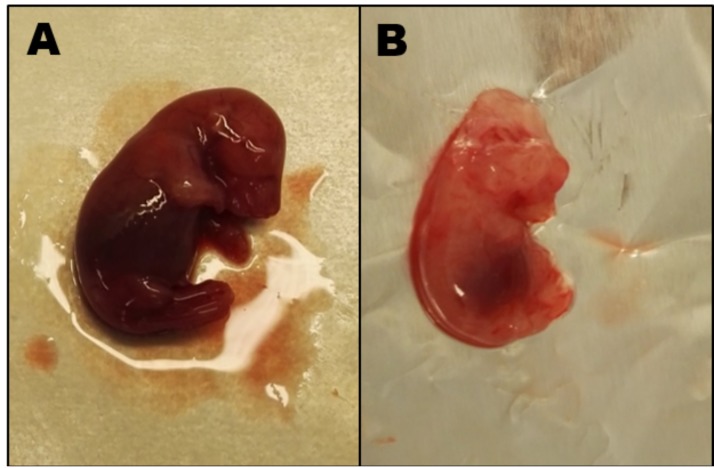
Day 19 rat fetuses. (**A**) Fetus from a 1C balanced rat mother weight 2.9 g. (**B**) Fetus from a 7-week 1C imbalanced rat mother showing cranial malformation and general visual underdevelopment, weight 1.3 g.

The basic characteristics of the four rat groups are shown in Table 2. During pregnancy, all maternal organs increased in weight, most noticeable the liver which increased to almost twice the weight of non-pregnant rats (*p* = 0.0025). The placentas show no difference between 1C balanced and 5-week 1C imbalanced rats, however, compared with the latter, the placentas from the 7-week 1C imbalanced rats weighed almost 25% less (*p* = 0.0076) (Table 2). In agreement with this, the fetus weight of 7-week 1C imbalanced dams was lower (median (range) 1.3 (0.9–2.1) g) than both 5-week 1C imbalanced fetuses (2.0 (1.6–3.41) g, *p* = 0.001) and 1C balanced fetuses (2.9 (1.9–3.4) g, *p* = 0.0001). The litter size was slightly lower in the 1C balanced rats (12 fetuses/litter) compared with the 5-week 1C imbalanced (14 fetuses/litter) and 7-week 1C imbalanced (15 fetuses/litter) rats (Table 2), which suggesting that neither fertility nor fertilization was affected by the 1C imbalanced diet. However, the number of living fetuses was far lower in the 7-week 1C imbalanced rats (<15%) than in the 5-week 1C imbalanced (~70%) and 1C balanced rats (>95%). As shown in Figure 2, there were visible changes in the morphological development in fetuses from 1C balanced and 7-week 1C imbalanced mothers. No visual malformations were observed for the 5-week 1C imbalanced fetuses (data not shown).

### 3.2. Distribution of Endogenous B12

We measured the content of endogenous B12 in plasma and in selected organs (Table 3). At baseline, there was no difference in plasma B12 between the two groups of 1C balanced rats (non-pregnant vs. pregnant) or between the two groups of 1C imbalanced rats (5-week and 7-week 1C imbalanced). However, plasma B12 was less than half in the 1C imbalanced rats (low B12 intake) compared with the 1C balanced rats (high B12 intake) (*p* < 0.0002). During pregnancy, all rats showed a significant decrease in plasma B12, and at late pregnancy (gestation day 19) plasma B12 was ~2 fold lower in the 1C balanced pregnant rats and ~6 fold lower in the 1C imbalanced rats compared with the non-pregnant rats (Table 3).

First, we compared the concentrations of B12 expressed per gram of tissue (Table 3). The most dramatic changes were found in the kidneys. In 1C balanced animals, pregnancy induced a reduction in kidney B12 to around half the values observed in non-pregnant rats (*p* = 0.0025), with an additional decrease in the 1C imbalanced groups reaching an almost 90% reduction in the 7-week 1C imbalanced pregnant rats (*p* = 0.0002). In contrast, pregnancy did not induce any changes in the B12/g content in liver or brain (compared with non-pregnant rats); whereas all organs from 1C imbalanced rats showed decreased values of endogenous B12 compared with 1C balanced rats (Table 3).

In the fetal compartment (uterus, placenta, fetuses), both uterus and placenta had significant lower B12/g when comparing 1C imbalanced rats with 1C balanced rats (placenta, *p* = 0.0002; uterus, *p* = 0.0164), whereas no difference was observed between 5-week 1C imbalanced and the 7-week 1C imbalanced mice. The endogenous B12/g in fetuses differed between 1C balanced and 1C imbalanced fetuses (*p* = 0.0107), while no difference was seen between the two 1C imbalanced groups (Table 3).

**Figure 3:**
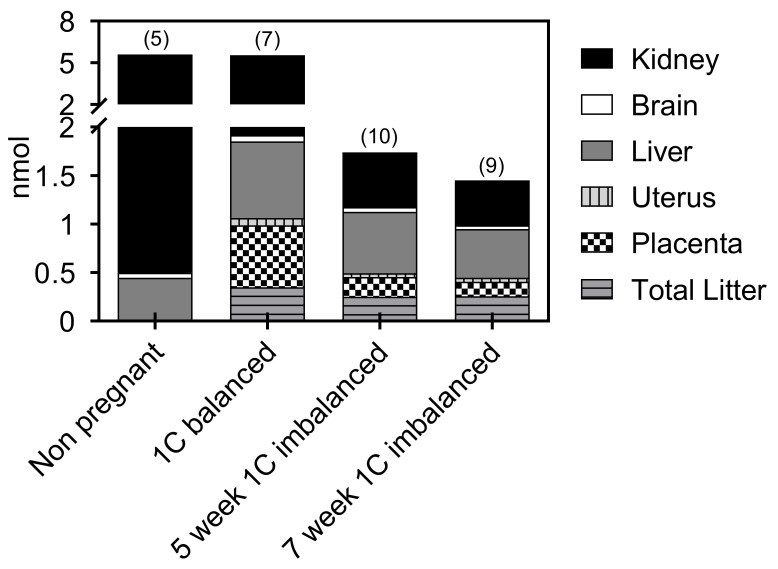
The sum of endogenous B12 recovered. Total amount of endogenous B12 in tissues from non-pregnant 1C balanced rats and from 1C balanced and 1C imbalanced pregnant rats at gestation day 19. The amount of B12 in each tissue was calculated by multiplying B12/g and the total organ weight. Results are given as stacked mean values (pmol). Number (*n*) of animals in each column is shown in parentheses.

Next, we explored the total organ content of B12 as depicted in Figure 3. We found no difference in total B12 accumulation between 1C balanced non-pregnant and pregnant rats (~5 nmol). The decreased content of B12 in the kidney of the pregnant rats was counteracted by an increased B12 accumulation in the liver and fetal compartment. The total amount of B12 that accumulated in the tissues of the 1C imbalanced rats were close to half of the values found in the 1C balanced rats and declined with the degree of B12 depletion. All organs showed lower values, but the largest differences were seen in the kidneys and the placenta. The 7-week 1C imbalanced mothers showed lower total B12 accumulation than the 5-week 1C imbalanced mothers (*p* = 0.01).

### 3.3. Distribution of Newly Absorbed Labeled B12

**Figure 4:**
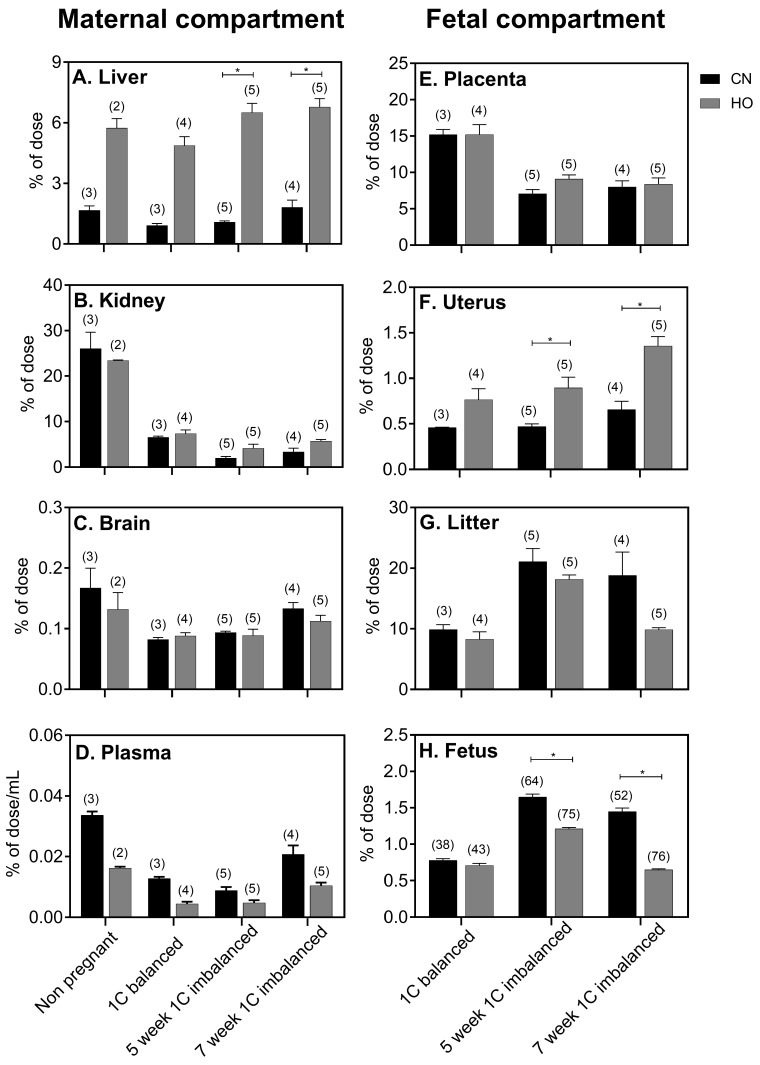
Labeled B12 in maternal tissues and fetuses. Tissue [^57^Co]B12 accumulation (CN-B12 (CN) or HO-B12 (HO)) in 1C balanced and 1C imbalanced rats at gestation day 20, measured 24 h after oral administration. Depicted are fractions of the administered [^57^Co]B12 (% of dose) present in selected organs per whole organ and as % of dose/mL plasma. Results are shown as mean with SEM. Number (*n*) of animals in each column is shown in parentheses. Differences between administrations of [^57^Co]CN-B12 (black) and [^57^Co]HO-B12 (grey) were estimated with the Mann–Whitney test with Bonferroni corrections (level of significance for maternal compartment (left column, **A**–**D**): *p* <0.05/4 = 0.01; level of significance for fetal compartment (right column, **E**–**H**), *p* <0.05/3 = 0.02). * Asterisks denote statistical significance according to the Bonferroni corrections.

In order to explore the B12 distribution 24 h after oral intake as a function of the form of the vitamin and the B12 status of the rats, we administered an oral trace dose of labeled B12 ([^57^Co]CN-B12 or [^57^Co]HO-B12). There was no difference in total mean [^57^Co]-B12 (both for [^57^Co]CN-B12 and (^57^Co]HO-B12) recovered (kidneys, liver, brain, fetal compartment) between non-pregnant (CN: 28%; HO: 29%) or pregnant 1C balanced rats (CN: 28%; HO: 27%). However, while the majority (CN: 93%; HO: 80%) of the recovered [^57^Co]-B12 was found in the kidneys of the non-pregnant rats, most (^57^Co]-B12 (CN: 89%; HO: 89%) was found in the fetal compartment (uterus, placenta, fetuses) of the pregnant rats. We recovered more [^57^Co]HO-B12 in the 5-week 1C imbalanced rats (HO: 39%) than in the 1C balanced rats (HO: 27%) (*p* = 0.007), but besides this there was no difference in total [^57^Co]-B12 recovered (both [^57^Co]CN-B12 and [^57^Co]HO-B12) between the three groups of pregnant rats (not demonstrated in figures). The distributions in the individual tissues are given in Figure 4. The distribution of [^57^Co]CN-B12 and [^57^Co]HO-B12 in the non-pregnant female rat showed similarity with the data previously reported for male rats [9] in that a relatively high fraction of [^57^Co]HO-B12 homed to the liver (Figure 4A), while higher amounts of [^57^Co]CN-B12 remained in the plasma (only an insignificant trend for female rats) (Figure 4D). In contrast to the studies in male rats [9], we did not see a significant higher accumulation of [^57^Co]CN-B12 in the kidney compared with [^57^Co]HO-B12 (Figure 4B).

The accumulation of [^57^Co]CN-B12 and [^57^Co]HO-B12 in the fetal compartment showed interesting differences. Higher amounts of [^57^Co]HO-B12 as compared to [^57^Co]CN-B12 homed to the uterus (*p* = 0.0159) (Figure 4F), whereas equal amounts of [^57^Co]-B12 were found in the placentas (Figure 4E) and in the fetuses (Figure 4H) of 1C balanced rats. The net transfer of [^57^Co]-B12 from mother to litter (Figure 4F) shows an insignificant trend to a higher accumulation of [^57^Co]CN-B12 than HO[^57^Co]-B12 in the 5-week 1C imbalanced and 7-week 1C imbalanced rats. When we look at the accumulation of [^57^Co]-B12 in the individual fetuses (Figure 4H), this trend becomes highly significant (5-week 1C imbalanced, *p* = 0.0001; 7-week 1C imbalanced fetuses, *p* = 0.0001) (Figure 4). Taken the two ways of handling the data together, we judge that more labeled CN-B12 is transported to the 1C imbalanced fetuses compared with labeled HO-B12.

## 4. Discussion

We investigated fetal outcome and endogenous B12 distribution and the accumulation of newly absorbed [^57^Co]CN-B12 and [^57^Co]HO-B12 in pregnant rats (and their fetuses) on 1C balanced and 1C imbalanced (low B12, high folate and high methionine) diets, respectively. We report that the 1C metabolism led to severe impact on macroscopic fetal development; that the distribution of endogenous B12 is influenced by both pregnancy and dietary components of the 1C metabolism; and that newly absorbed B12 is distributed differently depending on maternal B12 status and the B12 form administered. Furthermore, we find that HO-B12, in contrast to CN-B12, homes to the mother rather than the fetus, and that maternal B12 appears to reflect fetal outcome better than fetal B12.

Our study has some limitations. We only analyzed changes in B12 and did not include other components of the 1C metabolism. We analyzed selected maternal tissues, and thus cannot draw conclusions on the whole rat B12 distributions. Furthermore, the studies on 1C balanced rats was conducted three months prior to the 1C imbalanced rats, and the 7-week 1C imbalanced rats were two weeks older than the 5-week 1C imbalanced rats. However, with regard to baseline B12 levels, we do not have reason to believe that age would be of importance, as others have shown no variation in rat B12 according to age [16], and also we took great care in ensuring that the study conditions were identical for all groups (see Methods). Despite these weaknesses, we believe our data presents important new knowledge on maternofetal B12 accumulation during pregnancy, including the differences in trafficking of CN-B12 and HO-B12.

We introduced the rats to a 1C imbalanced diet with low B12, high folate, and high methionine during pregnancy starting two (5-week 1C imbalanced) or four (7-week 1C imbalanced) weeks prior to impregnation. The diet resulted in a B12 depletion in the rats. In humans, depletion of body B12 takes years [17]. However, here (and in our previous rat studies [9,10]), the levels of B12 decreased dramatically upon the relative short exposure to a low B12 intake. The decision to keep one group of rats on the 1C imbalanced diet for four weeks prior to impregnation (the 7-week 1C imbalanced group) was based on a previous study showing that this time period efficiently would induce a state of B12 deficiency [10]. However, we were concerned that severe 1C disturbances could prevent the rats from becoming pregnant, and thus we included a group of rats who was only kept on the 1C imbalanced diet for two weeks prior to impregnation (the 5-week 1C imbalanced group). However, in this study, the duration of the 1C imbalanced diet did not affect the rats’ ability to become pregnant. With regard to the low B12 content of the 1C imbalanced diet, this finding agrees with previous data showing that B12 deficiency in rats does not always affect litter size [18].

We measured the endogenous B12 in maternal tissues and fetuses. The sum of B12 recovered was the same for 1C balanced non-pregnant and pregnant rats, and the two groups absorbed an equal amount of the administered labeled B12. The results challenge the theory that B12 uptake is increased during pregnancy to meet the demands of the fetus and the pregnant body [19]. Instead, these demands seems to be met by a redistribution of B12 from the kidney to the liver and the fetal compartment.

Compared with the pregnant 1C balanced rats, the total amount of endogenous B12 recovered was reduced to one half and one third in the 5-week 1C imbalanced and 7-week 1C imbalanced rats, respectively. In the mothers, the reduction was most pronounced in the kidneys, but also the content in the liver was markedly reduced. In the fetal compartment, we found the placenta to show the greatest difference in B12 content, while the difference in fetal B12 content was less pronounced. This is in agreement with the general accepted theory that the placenta functions as a B12 storage organ, accumulates large quantities of B12 in times of vitamin surplus, and regulates the transport of B12 to the fetal department in a rate-limited manner, possibly by a receptor-mediated mechanism [11,12]. Our results for the newly-absorbed [^57^Co]-B12 further supports this hypothesis. In 1C balanced rats with high B12 status, the fetal/placental ratio is 55% (endogenous B12), but only 5% for the 24-h absorbed [^57^Co]CN-B12 and [^57^Co]HO-B12. In contrast, these numbers are 200% (endogenous B12), 18% [^57^Co]CN-B12, and 8% [^57^Co]HO-B12 for the 7-week 1C imbalanced (low B12) rats. In continuation of this, the 5-week 1C imbalanced rats shows intermediate values (103% (endogenous B12), 24% [^57^Co]CN-B12, and 14% [^57^Co]HO-B12) between the 1C balanced and 7-week 1C imbalanced groups. These figures suggests that more B12 is transported from the placenta to the fetus in times of B12 depletion. Ullberg et al. (1967) [11] and Graber et al. (1971) [12] suggest that a receptor-mediated mechanism is at play for the placental–fetal B12 transfer. In the Ullberg study, they found a high concentration of radiolabeled CN-B12 in the placenta 15 min after intravenous injection to 20-day pregnant rats, with no presence of labeled B12 in the fetus. Within the next days, B12 was slowly transferred to the fetus eventually leading to 40 to 130 times higher concentrations per gram tissue in the fetuses compared with the mother. This pattern is unique for B12, as the placenta does not accumulate other micronutrients, such as vitamin A, vitamin B1, vitamin C, vitamin E, amino acids, glucose, iron, and iodine [11]. Another possibility is that the placenta is enriched in B12 binding proteins and that the differences in placental B12 accumulation between the dietary groups is a matter of differences in the B12 saturation level.

Though we did not aim at studying the influence of the 1C imbalanced diet on fetal development, we observed that 7 weeks on this diet resulted in visual underdevelopment, which was not seen in the 5-week 1C imbalanced (or 1C balanced) fetuses. This was mirrored in the B12 accumulation of the mothers, but surprisingly not in the B12 accumulation of the fetuses. There was no difference in the amount of endogenous B12 between the 5-week 1C imbalanced and the 7-week 1C imbalanced fetuses. We take this to indicate that maternal B12, rather than fetal B12, is important for fetal outcome. This in turn may explain the paradox that a poor maternal B12 status is related to impaired fetal outcome [1,2,3,4,5], while children with severe B12 deficiency caused by inborn errors in the B12 machinery may be without symptoms at birth [7]. Another likely possibility is that the fetal underdevelopment is a result of other components in the 1C imbalanced diet. Excess folate alone has previously been shown to cause placental abnormalities and embryonic defects in mice [20,21]. In addition, low B12 combined with high folate may exacerbate B12 deficiency [21,22]. The 1C imbalanced diet contained large quantities of methionine (10,635 mg/kg diet), which has been shown to cause in poor physiological and pathological outcomes, both alone and combined with low B12 [23,24]. The high levels of choline chloride (1012 mg/kg), glycine (3030 mg/kg), serine (5130 mg/kg) and valine (3170 mg/kg) may also affect the 1C metabolism (and indirectly the B12 metabolism) in different harmful ways [23]. From this perspective, future studies are requested to clarify to which extend lack of B12 per se is the causative agent for a poor fetal outcome.

The 1C balanced (high B12) fetuses accumulated comparable amounts of [^57^Co]CN-B12 and [^57^Co]HO-B12. Surprisingly, this was not the case for fetuses of 1C imbalanced (low B12) dams. In these fetuses, [^57^Co]CN-B12 accumulated to a higher degree than HO[^57^Co]-B12, and the largest difference was observed in the fetuses of 7-week 1C imbalanced rats. We do not have a firm explanation for this observation, but speculate that it is driven by the preferential uptake/retention of [^57^Co]HO-B12 by maternal organs, thereby leaving a surplus of CN-B12 for the fetus. Naively, one could take this finding to indicate that CN-B12 better than HO-B12 would rescue the B12 status of the fetus. However, before such a conclusion can be drawn, we need more knowledge on the fetal metabolism of CN-B12. In order to play an active role in the cells, both CN-B12 and HO-B12 must be converted into active co-enzyme forms. Previous studies in rats and in vitro suggest a more efficient conversion of HO-B12 as compared to CN-B12 [25].

The rat (Rattus Norvegicus) is a well-known and important mammalian model, frequently used for studies on B12 metabolism. While this model allows us to study aspects of the metabolism of importance, also for humans, it is important to realize that the two species show important differences in the B12 metabolism. While humans respond to deficiency with symptoms from both the hematological and neurological systems, few signs of B12 deficiency has been reported in the rat. Because of such differences, our results cannot be directly translated into the human setting. However, we find that the results offers a key to understand the current puzzle that B12 deficiency has consequence both for mother and child, while a child with an inborn ability to utilize B12 may be born without symptoms by a B12 replete mother. Thus, clinical studies focusing on whether fetal or maternal B12 status is the most important is warranted, and in this context, it will be important to clarify whether CN-B12 or HO-B12 should be recommended for supplementation during pregnancy.

In conclusion, we confirm that keeping rats on a 1C imbalanced diet containing a low supply of B12 but an increased supply of folate and methionine impairs fetal development. We report details on the B12 distribution between mother and fetus as a function of maternal B12 status. Notably, we find that HO-B12 homes to the mother rather than the fetus, and that maternal B12 accumulation appears to influence fetal outcome more than does fetal B12 accumulation.

## Figures and Tables

**Table 1:** Dietary composition of the 1C balanced diet and the 1C imbalanced diet. Compared with the 1C balanced diet (standard rat diet) (Altromin 1324 from Brogaarden, https://altromin.com/pdf/en/1320), the 1C imbalanced diet (Altromin C1024 fra Brogaarden, https://altromin.com/pdf/en/C1024) contained half the amount of B12 and was higher in content of folate, B6, choline chloride, methionine, glycine, and serine.

Units Nutrients/kg Diet	1C Balanced Diet (Altromin 1324)	1C Imbalanced Diet (Altromin C1024)	Ratio of C1024 Content Compared to 1324 Content
Protein, kcal (%)	768 (24%)	680 (18%)	0.9
Carbohydrate, kcal (%)	2091 (65%)	2705 (70%)	1.3
Fat, kcal (%)	367 (11%)	453 (12%)	1.2
Vitamin B12 (CN-B12), µg	24	11	0.5
Folic Acid, mg	2	10	5
Vitamin B6, mg	9	15	1.7
Choline Chloride, mg	600	1012	1.7
Methionine, mg	0.3	10,635	35,450
Glycine, mg	0.8	3030	3788
Serine, mg	0.9	5130	5700

**Table 2:** Basic characteristics. 1C balanced and imbalanced rats and their offspring at pregnancy day 19 out of 21.

	1C Balanced Rats		1C Imbalanced Rats	
	Non-Pregnant *n* = 5	Pregnant *n* = 7	5-Week 1C Imbalanced *n* = 10	7-Week 1C Imbalanced *n* = 9
Age at sacrifice (week)	12	12	12	14
Weight of rats, at study start, D1 (g)	235 (212–239)	228 (210–242) ^^^	264 (247–291) *	312 (282–326) *^,+^
Weight of rats, at sacrifice, D19 (g)	270 (238–279)	373 (349–395) ^^^	416 (395–462) *	420 (362–474) *
Litter size (*n*)	X	12 (7–14)	14 (12–17) *	15 (7–18) *
Single fetus weight (g)	X	2.9 (1.9–3.5)	2.0 (1.6–3.1) *	1.3 (0.9–2.1) *^,+^
Maternal organs				
Kidneys (g)	2.2 (2.1–2.4)	2.5 (2.3–3.4) ^^^	3.0 (2.7–3.2) *	3.2 (2.7–3.5) *
Liver (g)	9.3 (7.9–9.8)	16.1 (14.2–17.2) ^^^	18.1 (15.9–19.4)	16.1 (13.7–19.2)
Brain (g)	2.1 (1.8–2.4)	2.4 (2.2–2.6) ^^^	2.5 (2.3–2.8)	2.4 (2.1–2.5)
Uterus (g)	0.8 (0.7–1.4)	6.8 (4.2–9.7) ^^^	5.4 (4.0–7.1)	6.6 (3.8–9.2)
Placenta (g)	X	7.9 (5.3–10.1) ^^^	8.0 (7.6–9.2)	6.2 (3.8–8.1) ^+^

Results are presented as median with (range). Significant differences (*p* ≤ 0.05) are denoted as follows: non-pregnant vs. 1C balanced pregnant rats (^^^); 1C balanced vs. 5-week 1C imbalanced pregnant rats (*); 1C balanced vs. 7-week 1C imbalanced pregnant rats (*); and 5-week 1C imbalanced vs. 7-week 1C imbalanced pregnant rats (^+^). X = No data. D1: baseline, D19: Gestational day 19 for pregnant rats.

**Table 3:** Endogenous B12 in maternal tissues and fetuses. B12 content in plasma and organs from 1C balanced and 1C imbalanced rats before pregnancy (only plasma) and at gestation day 19.

	1C Balanced Rats		1C Imbalanced Rats	
	Non-Pregnant *n* = 5	Pregnant *n* = 7	5-Week 1C Imbalanced *n* = 10	7-Week 1C Imbalanced *n* = 9
Plasma D1 (pmol/L)	1322 (1191–1422)	1241 (947–1313)	498 (355–593) *	483 (412–625) *
Plasma D19 (pmol/L)	1247 (1105–1321)	727 (643–923) ^^^	217 (159–287) *	208 (166–328) *
Organ B12 levels G19				
Kidneys (pmol B12/g)	2201 (2086–2649)	1276 (1149–1708) ^^^	190 (150–274) *	148 (108–183) *^,+^
Liver (pmol B12/g)	47 (44–51)	48 (25–74)	35 (26–52)	30 (26–41) *
Brain (pmol B12/g)	24 (22–24)	27 (23–33)	19 (16–24) *	16 (13–21) *^,+^
Uterus (pmol B12/g)	13 (10–17)	12 (4–15)	7 (4–9) *	6 (4–9) *
Placenta (pmol 12/g)	x	75 (61–99)	25(18–36) *	22 (14–38) *
Fetus (pmol B12/g)	x	29 (27–31)	18 (11–23) *	17 (14–22) *

Results are presented as median with (range). Significant differences (*p* ≤ 0.05) are denoted as follows: non-pregnant vs. 1C balanced pregnant rats (^); 1C balanced vs. 5-week 1C imbalanced pregnant rats (*); 1C balanced vs. 7-week 1C imbalanced pregnant rats (*); and 5-week 1C imbalanced vs. 7-week 1C imbalanced pregnant rats (^+^). X = No data. D1: baseline, D19: Gestational day 19 for pregnant rats.
